# Eye-Tracking-Based Analysis of Situational Awareness of Nurses

**DOI:** 10.3390/healthcare10112131

**Published:** 2022-10-26

**Authors:** Masahiro Sugimoto, Atsumi Tomita, Michiko Oyamada, Mitsue Sato

**Affiliations:** 1 Institute of Medical Sciences, Tokyo Medical University, Shinjuku, Tokyo 160-0022, Japan; 2 Institute for Advanced Biosciences, Keio University, Tsuruoka 997-0052, Japan; 3 Department of Nursing, Nihon Institute of Medical Science, Moroyama 350-0435, Japan; 4 Department of Nursing, Kiryu University, Midori 379-2392, Japan

**Keywords:** eye tracking, nursing, education

## Abstract

Background: Nurses are responsible for comprehensively identifying patient conditions and associated environments. We hypothesize that gaze trajectories of nurses differ based on their experiences, even under the same situation. Methods: An eye-tracking device monitored the gaze trajectories of nurses with various levels of experience, and nursing students during the intravenous injection task on a human patient simulator. Results: The areas of interest (AOIs) were identified in the recorded movies, and the gaze durations of AOIs showed different patterns between experienced nurses and nursing students. A state transition diagram visualized the recognition errors of the students and the repeated confirmation of the vital signs of the patient simulator. Clustering analysis of gaze durations also indicated similarity among the participants with similar experiences. Conclusions: As expected, gaze trajectories differed among the participants. The developed gaze transition diagram visualized their differences and helped in interpreting their situational awareness based on visual perception. The demonstrated method can help in establishing an effective nursing education, particularly for learning the skills that are difficult to be verbalized.

## 1. Introduction

Nursing education requires the efficient use of educational resources to teach relevant skills to students. In addition to lectures and practical training, licensed nursing requires learning based on the experiences of nurses and medical doctors.

Recently, information technology has been applied to realize efficient nursing education. The adoption of e-learning at various nursing institutes [1] has been further accelerated by the requirement of social distancing imposed by the COVID-19 pandemic. A web-based learning system has been utilized to facilitate interactions between students and teachers [2]. Moreover, a telemedicine-based simulation was developed to impart maternity nursing education [3]. Virtual reality has been utilized to simulate clinical event sequences to enrich the experience of medical students [4,5], which has enabled efficient training. Multimedia content, including web-based media, podcasts, and videos, has also been utilized for novel and efficient approaches to nursing education [6].

Eye-tracking is an educational methodology that has been applied to the medical [7,8,9] and healthcare fields [10]. Eye trails of medical clinicians were analyzed to understand their method of information extraction from electronic medical records that informed their decisions [11]. Eye-tracking was also used for a structural and objective examination of medical students in a clinical environment [12]. A visual inspection performed using an endoscope is critical to decision making during surgeries, and eye-tracking was implemented to estimate operators’ stress and fatigue levels during such procedures [13].

Eye-tracking has also been utilized to understand the process of knowledge and skill acquisition by professional nurses from their educators and more experienced colleagues [14]. Nursing errors depend on the situational awareness (SA) of nurses. The ability to recognize the current situation and predict situations soon—in this context, eye-tracking-based feedback has been conveyed to nurses to reduce such errors [15,16,17].

Here, we hypothesized that the gaze trajectory of nurses depends on their experience. Therefore, the objective of this study was to analyze the relationship between the nursing experience and eye-trajectory. We analyzed eye trails of experienced and novice nurses and nursing students using an eyeglass device with eye-tracking functions. A human patient simulator was used, and the participants were required to confirm the status of continuous infusion. An original diagram to depict the gaze transition and duration was developed to help the interpretation of situation awareness of participants.

## 2. Materials and Methods

### 2.1. Eye-Tracking

ViewTracker3 (DITECT Co., Ltd., Tokyo, Japan) was used to monitor eye trails in this study. This device has been utilized for tasks in various fields, e.g., the evaluation of consumers’ eye trails while extracting information from commercial labels on beef packages [18]. It is also used in the analysis of eye trails of teachers in classroom environments to design efficient education programs [19], optimization of warning message placements to ensure the safety of chemical experiments [20], and analysis of student’s eye trails while learning from e-learning content [21].

**Figure 1:**
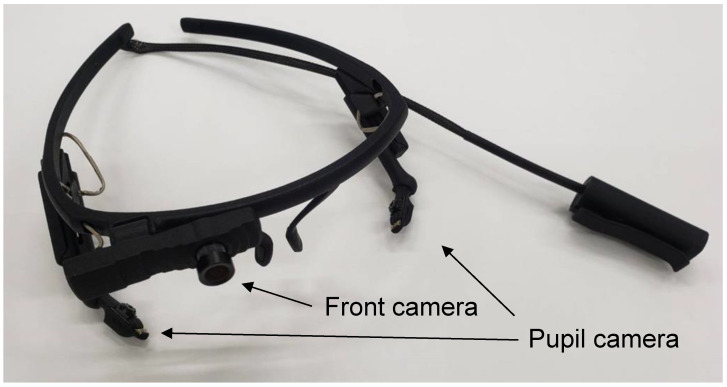
Wearable glasses for monitoring eye trails. The front camera captures frontal photographs, and the two-pupil cameras are used to track the pupils. The device is connected to a PC via a USB interface.

The architecture of ViewTracker3 is depicted in Figure 1. A camera was used to monitor the frontal surface of the face of the user, and the two cameras were used to monitor their pupils.

### 2.2. Study Design

**Figure 2:**
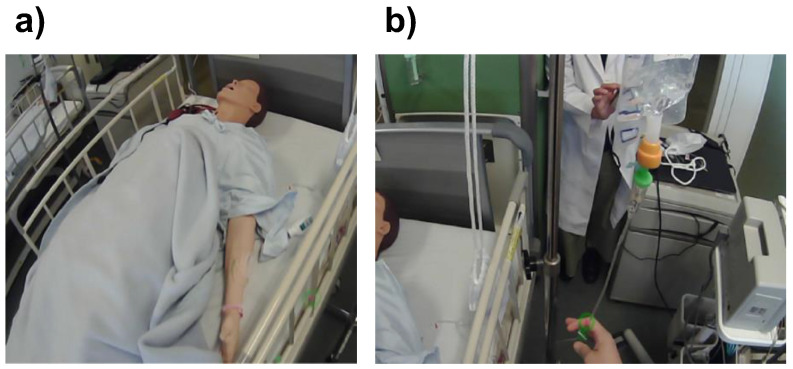
Environment for the eye-tracking tasks. (**a**) Human patient simulator and (**b**) continuous infusion.

Three licensed nurses with different degrees of experience and two nursing students from different grades were enrolled in this study (Table 1). The human patient simulator, Nursing Anne (Laerdal Medical, Stavanger, Norway), was used to simulate identical environments for all participants. A continuous infusion was selected as the target task because all participants were acquainted with it (Figure 2). Participants were given the following instructions before the execution of the assigned task. “The patient underwent surgery two days ago. A continuous infusion has been mounted. Please check the site of intravenous insertion for any problem, e.g., redness or swelling. The speed of the intravenous drip should be adjusted to 80 mL/h. An adequate consciousness level has already been confirmed. Please confirm the status of continuous infusion.”

### 2.3. Data Collection

Before each participant performed the task, the initialization of eye trails was adjusted using a calibration program. To this end, a circle was displayed at the center of a monitor connected to the device, and, subsequently, at each corner of the monitor sequentially. Each participant was instructed to gaze at the circles without moving their face. The consistency between the actual gaze and monitored points on the monitor was confirmed by instructing each participant to gaze at four or five points at distances of 2–3 m. In the case of insufficient consistency, the calibration process was repeated.

ViewTracker3 was configured as follows. A front camera with a resolution of 1280 × 720 pixels and a frame rate of 30 Hz was used. Two pupil cameras with resolutions of 192 × 192 pixels and frame rates of 120 Hz were used. Furthermore, the edge intensity (dimensionless) was considered to be 23, eye size (dimensionless) was considered to lie in 10–150, and sensitivity for the detection of the black iris (dimensionless) was selected to be 0.997. The software, ViewTracker3 (ver. 1.0520, DITECT, Tokyo, Japan), was used with default configurations in high-resolution mode (M-JPEG).

The device was connected using a USB3 interface to a PC with the following configurations: operating system: Windows 64bit Pro, CPU: Intel^®^Core™i7-8650U CPC@1.90GHz 2.11GHz, memory: 16.0GB DDR4 2400 MHz, monitor: 12.5 inches (1920 × 1080 pixels), and storage: SSD:512GB M.2 2280 PCIe.

#### Ethical Considerations

This study was performed in accordance with the Declaration of Helsinki principles. The study protocol was approved by the Ethics Committee of Kiryu University (no. 2022-02). Written informed consent was obtained from each participant prior to participating in the study.

### 2.4. Data Analysis

Corresponding to each participant, the AOIs—regions where their eye trail halted—were identified. Two researchers analyzed the movies. The identification of AOIs was manually performed and reviewed by others. Both the transition of the tire trail and the gaze duration were visualized using a custom state transition diagram (STD). A standard STD was customized to visualize transitions of both eyes over the AOIs and the gaze duration corresponding to each AOI. Clustering analysis of the gaze duration was performed using Spearman correlation to analyze the similarity in participants’ gaze duration patterns. MeV TM4 (ver.4.9.0) was used for this purpose (https://sourceforge.net/projects/mev-tm4/, accessed on 1 Mar 2022).

## 3. Results

### 3.1. Determination of AOIs and Customization of STD

**Figure 3:**
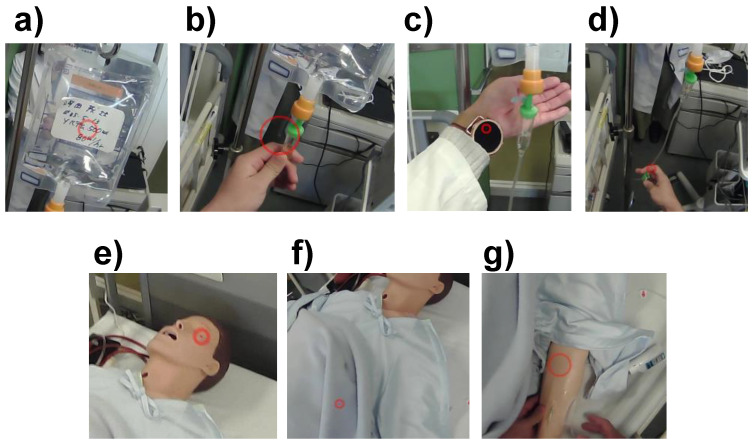
AOIs in this study: (**a**) bag, (**b**) chamber, (**c**) watch, (**d**) cock, (**e**) face, (**f**) sheets, and *(***g**) inner elbow.

Figure 3 depicts seven AOIs determined based on the sufficient gaze durations during the assigned task—namely, the bag, chamber, watch, cock, face, sheets, and inner elbow. The eye trails were observed to shift between the watch and chamber frequently, and a single AOI, i.e., watch, was defined.

**Figure 4:**
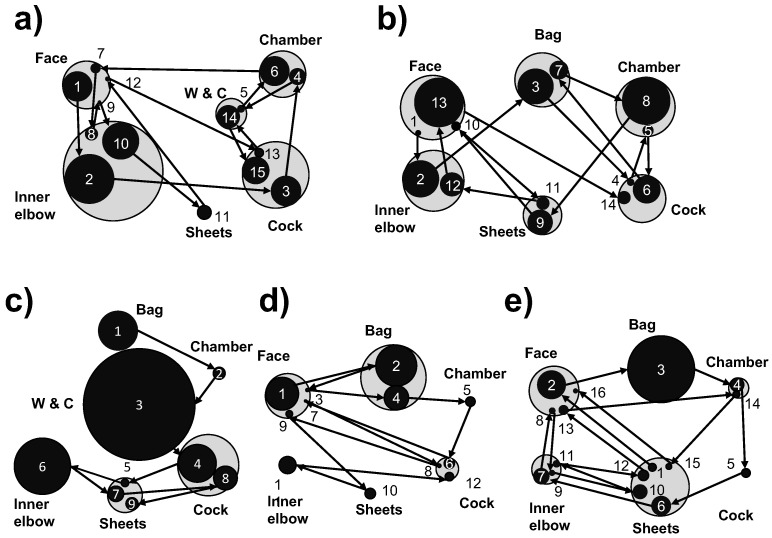
Gaze transition between the AOIs. The circles and arrows represent AOIs and transitions between pairs of AOIs, respectively. The numbers indicate the order of transition. The black and gray circles indicate the gaze duration corresponding to an AOI and the total gaze duration corresponding to an AOI, respectively. Panels (**a**–**e**) correspond to the data gathered from five participants, nurses 1–5. W & C denotes watch and chamber, respectively.

A customized STD was designed to visualize eye transition patterns, as depicted in Figure 4. In the figure, the relationships between the various fixed duration of AOIs and eye transitions are visualized simultaneously. The black circles indicate the durations of individual AOI gazes. The gray circles indicate the total duration of gazes corresponding to an AOI.

Figure 4a depicts the gaze transition of the most experienced licensed nurse. Their gaze trail exhibited the following sequence: (1) a gaze at the face of the human patient simulator and (2) a gaze of longer duration at the inner elbow. Subsequently, the gaze shifted to evaluate the configurations and readings of intravenous devices, including (3) the cock, (4) chamber, (5) chamber and watch, and (6) chamber. Then, the gaze returned to the patient simulator, and it rested on the face repeatedly while switching to other parts in the following order: (7) the face, (8) inner elbow, (9) face, (10) inner elbow, (11) sheets, and (12) face. The observations confirmed that the gaze was repeatedly moved to the face at the beginning and end of the task.

Figure 4b depicts the gaze transition of the licensed nurse with the second-highest experience (10 y). Initially, the gaze was observed to be directed at (1) the face and (2) inner elbow. Subsequently, the gaze moved to the intravenous devices (3)–(8), and back to simulators (9)–(12).

Figure 4c depicts the transition of gaze trajectory of the novice nurse with 8 months of experience. In this case, the gaze did not confirm the status of the simulator initially—it was directed at (1) the bag, (2) chamber, and (3) chamber and watch, in sequence. In particular, the gaze duration corresponds to (3) the chamber and the watch. Furthermore, the nurse did not gaze at the face of the simulator; instead, a longer duration was spent to stare at (6) the inner elbow.

Figure 4d illustrates the data of a nursing student in the 4th grade. In this case, the gaze sequentially progressed along (1) the face, (2) bag, (3) face, (4) bag, (5) chamber, (6) cock, and (7) face.

Figure 4e presents the data of a nursing student in the 3rd grade. In this case, the gaze sequentially progressed along (1) the sheets, (2) face, (3) bag, (4) chamber, (5) cock, back to the simulator, (6) sheets, (7) inner elbow, (8) face, (9) inner elbow, and (10) sheets. In the final stage, the gaze was moved to confirm the overall situation of the simulator.

### 3.2. Differences between Gaze Durations Corresponding to Different AOIs

**Figure 5:**
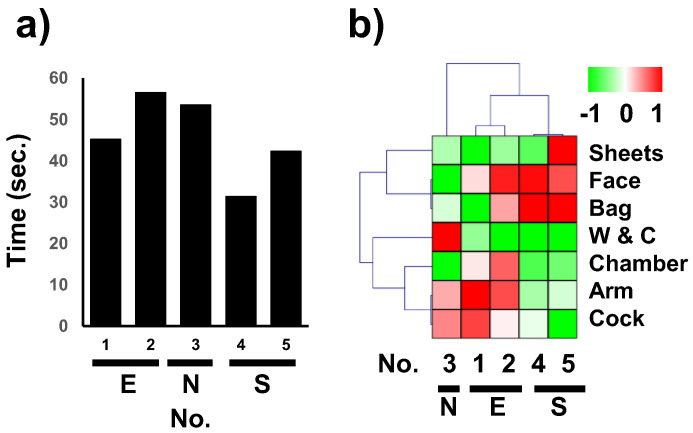
Durations corresponding to AOIs: (**a**) Total gaze duration of all AOIs throughout the task. (**b**) Clustering of gaze durations corresponding to different AOIs. For each participant, the gaze duration time was normalized to calculate the Z-score, and clustering was performed using Spearman rank correlation. The color bar on the right indicates the Z-score—red, green, and white denote relatively longer, shorter, and average durations, respectively. E, N, and S denote experienced, novice, and student nurses, respectively. W & C denotes watch and chamber, respectively.

Figure 5a depicts the total gaze durations corresponding to the AOIs. The gaze duration of the three licensed nurses is observed to be longer than those of the two nursing students.

Figure 5b depicts a heatmap of the relative duration corresponding to each AOI. As depicted in the figure, clustering analysis results revealed similarities between the gaze directions corresponding to participants as well as AOIs. The novice licensed nurse (nurse 3) accounted for the largest deviation in the cluster dendrogram, which indicated that their gaze followed a pattern that was the most different compared to those of the other four participants—the gaze durations of nurse 3 corresponding to the watch and the chamber AOIs were significantly longer compared to the other AOIs, and this trend was also significantly different from the gaze characteristics of the other participants. The gaze characteristics of experienced nurses (nurses 1 and 2) and nursing students (nurses 4 and 5) were in agreement with each other. The three licensed nurses spent relatively longer durations gazing at the inner elbow and the cock compared to the other AOIs. Moreover, the two nursing students spent longer durations gazing at the bag and face, and shorter durations at the other AOIs, except for the sheets in the case of nurse 5.

## 4. Discussion

In this study, the eye trails of nurses with various levels of experience were recorded and visualized using a customized STD and a heatmap representation based on clustering analysis. Analysis of visual information using computational techniques, such as artificial intelligence, is an active topic of research, and several such applications have already been published. For example, the diagnosis of breast cancer based on mammography data via image processing requires the analysis of static images [22]. The analysis of surgeries using endoscopes requires data processing on the video and dynamic images recorded using the endoscope. However, the current study demonstrated that additional images may also be essential to computational processing in a clinical environment—gaze tracking of nurses confirmed that, besides the inner elbow, images of the face of the patient and the intravenous drip device were also important to confirm their status and gather a comprehensive understanding of the situation. It is difficult to teach such skills through textbooks, and the related proficiency levels are dependent on individual expertise and experience. Therefore, quantitative and objective analyses to analyze the differences between the eye trails of experienced and novice nurses can be expected to yield a comprehensive educational tool.

The total gaze durations recorded in this study revealed that the two nursing students spent less time on the given task compared to the licensed nurses (Figure 5a). Cognizance of this result may help nursing students to perform their tasks more efficiently. The durations corresponding to different AOIs corresponding to the two nursing students were similar, as were those corresponding to the two experienced nurses (Figure 5b).

According to the recorded eye trail data, one of the experienced nurses (nurse 2) spent a longer duration gazing at (8) the chamber after gazing at (6) the cock and (7) bag, indicating that they adjusted the intravenous drip speed without gazing at the watch (Figure 4b). The other licensed nurses also adjusted the intravenous drip speed (Figure 4a,c). However, both nursing students skipped this step, even though their gaze moved to the bag and chamber (Figure 4d,e). This represents a misunderstanding of a patient’s situation, and its visualization using the customized STD is expected to teach nursing students the proper methodology to evaluate a patient’s situation.

An eye-tracking comparison between experienced nurses and nursing students indicated that the gaze duration of the experienced nurses corresponding to the puncture point before the intravenous injection was longer than that of the nursing students, which implied that the former paid more attention to confirming SA before the task [14]. AI-Moteri et al. reviewed the application of eye-tracking studies in medical fields and classified errors in decision-making into three primary categories—detection, recognition, and judgment errors [8]. In the current study, the gaze durations of the two student nurses corresponding to the chamber and the bag were short, and did not adjust the drip speed (Figure 4d,e); therefore, this can be categorized as a recognition error. The two experienced nurses frequently gazed at the face of the simulator, which minimized errors of all three types (Figure 4a,b). Thus, the use of a simulator in response to unexpected treatments is expected to be suitable for vital confirmation.

This study suffered from several limitations. Multiple studies have analyzed nurses’ eye trails. In [23], the eye trails of scrub nurses with various levels of experience were analyzed to understand the relationship between attention management strategies and performance. Moreover, the eye trails of three nursing students were analyzed [17] to determine embedded mislabeled patient identification numbers. Tad et al. reviewed the eye-tracking-based diagnostic interpretation of medical imaging and classified visual behavior patterns into nine categories [24]. These categories included (1) fixation count, i.e., the number of gazes at an AOI; (2) regressive fixation count, i.e., recursive gaze movements between two AOIs; (3) fixation duration, i.e., the periods of gaze at an AOI; (4) amplitude, i.e., the magnitude of a saccade; (5) saccade peak velocity, i.e., the speed of gaze movement between AOIs, and four other categories, e.g., the blink rate of eyes. In the current study, blink data were not collected and categories (4) and (5) were not analyzed. A study on the eye-tracking of nurses during the monitoring of electrocardiograms claimed that a high number of participants were necessary to draw an objective evaluation in their trials [25]. The low number of participants in this study is one of its limitations. Although the development diagram indicated a difference in gaze transitions among the participants, experiments with independent participants are necessary for validating the methodology. There are still technical problems to be improved. The eyeglass devices used in this study required the adjustment of pupil cameras for tracking eye motions. Furthermore, initialization of the gaze position was required. The participants were repeatedly required to repeat these processes until the eyeglasses accurately captured the eye trajectory. These processes are more time-consuming when compared with the substantive nursing care experiments in this study, thereby limiting the number of experiments. Automated data analysis using the recorded movie should also be implemented to establish efficient feedback for each participant. Furthermore, the identification of AOI should be automated for a more objective evaluation of gaze transitions.

The establishment of effective nursing education by integrating conventional training and eye-tracking technology is challenging. Debriefing after practical training is a common method in nursing education. One of the methods to establish effective nursing education is the visualization of the difference in eye trajectories depending on the participant’s experience. Henneman et al. compared the feedback methods of nursing students using simulation-based practice and found that eye-tracking-based feedback was more effective than verbal debriefing [26]. Therefore, the effectiveness of customized STD in training nurses should be evaluated in future studies.

## 5. Conclusions

This study hypothesized that gaze trajectories of nurses differ based on their experiences, even under the same situation. Therefore, we analyzed the eye movements of nurses with various levels of experience and two nursing students on a human simulator. Wearable eyeglasses with frontal and pupil cameras were used to monitor gaze trajectories. The confirmation of continuous infusion status in a human patient simulator was selected as the task. Clustering analysis of the gaze durations corresponding to different AOIs revealed a high degree of similarity between the two experienced nurses and two nursing students. Visualization of the eye movement patterns using a customized STD differentiated the licensed nurses from the nursing students and revealed more characteristics. For example, the adjustment of the intravenous drip speed and confirmation of vital signals by repeatedly gazing at the face of the simulator were tasks that differentiated nurses of the two groups. The conclusions of this study are expected to facilitate the understanding of empirically obtained skills and tacit SA of experienced nurses, which is difficult to teach using a textbook. Therefore, eye-tracking may be used as a complementary educational tool.

## Figures and Tables

**Table 1:** Characteristics of the participants.

**No.**	**Age**	**Sex**	**Experience**	**Note**
1	52	F	26Y	Experienced
2	30	M	10Y	Experienced
3	23	F	8M	Novice
4	20	F	3rd grade	Student
5	22	M	4th grade	Student

## Data Availability

The data presented in this study are available on request from the corresponding author.
